# Bone marrow mesenchymal stem cells protect against *n*-hexane-induced neuropathy through beclin 1-independent inhibition of autophagy

**DOI:** 10.1038/s41598-018-22857-x

**Published:** 2018-03-14

**Authors:** Jie Hao, Shuangyue Li, Xiaoxia Shi, Zhiqiang Qian, Yijie Sun, Dunjia Wang, Xueying Zhou, Hongxin Qu, Shuhai Hu, Enjun Zuo, Cong Zhang, Liyan Hou, Qingshan Wang, Fengyuan Piao

**Affiliations:** 1 Department of Occupational and Environmental Health, Dalian Medical University, Dalian, Liaoning 116044 China; 2 College of Stomatology, Dalian Medical University, Dalian, Liaoning 116044 China; 3 Department of Nutrition and Food Safety, Dalian Medical University, Dalian, Liaoning 116044 China

## Abstract

Chronic exposure to *n*-hexane, a widely used organic solvent in industry, induces central-peripheral neuropathy, which is mediated by its active metabolite, 2,5-hexanedione (HD). We recently reported that transplantation of bone marrow-mesenchymal stem cells (BMSC) significantly ameliorated HD-induced neuronal damage and motor deficits in rats. However, the mechanisms remain unclear. Here, we reported that inhibition of HD-induced autophagy contributed to BMSC-afforded protection. BMSC transplantation significantly reduced the levels of microtubule-associated protein 1 light chain 3-II (LC3-II) and the degradation of sequestosome-1 (p62) in the spinal cord and sciatic nerve of HD-intoxicated rats. Downregulation of autophagy by BMSC was also confirmed in VSC4.1 cells exposed to HD. Moreover, inhibition of autophagy by PIK III mitigated the neurotoxic effects of HD and, meanwhile, abolished BMSC-afforded neuroprotection. Furthermore, we found that BMSC failed to interfere with Beclin 1, but promoted activation of mammalian target of rapamycin (mTOR). Unc-like kinse 1 (ULK1) was further recognized as the downstream target of mTOR responsible for BMSC-mediated inhibition of autophagy. Altogether, BMSC transplantation potently ameliorated HD-induced autophagy through beclin 1-independent activation of mTOR pathway, providing a novel insight for the therapeutic effects of BMSC against *n*-hexane and other environmental toxicants-induced neurotoxicity.

## Introduction

Chronic exposure to organic solvent, *n*-hexane results in central-peripheral neuropathy, which is mainly mediated by its toxic metabolite, 2,5-hexanedione (HD)^1^. Clinical patients of *n*-hexane-induced neuropathy display sensor-motor deficits, such as numbness and tingling sensation in the toes and fingers as well as weakness in distal legs^2^. Morphological studies revealed that axon atrophy, segmental demyelination and distal axonal Wallerian degeneration are the main pathological damage^3^. Due to the wide use of *n*-hexane in various industrial processes, *n*-hexane-related neurotoxicity gradually becomes a major health concern and occupational health hazard in exposed human. However, no effective treatment is available currently.

Bone marrow mesenchymal stem cells (BMSC) are adult stem cells with strong self-renewing and multilineage differentiation abilities^4^. Due to easily obtained and displaying potent anti-inflammatory, anti-apoptotic capacities and neurotrophic functions, BMSC gain much attention and have been used to treat various diseases^5^. The beneficial effects including neuroprotection and/or improved functional recovery, by BMSC transplantation have been demonstrated in multiple neurological pathologies, such as spinal cord injury, ischemic stroke, Alzheimer’s disease, Parkinson’s disease and Amyotrophic Lateral Sclerosis^6–8^. Recently, we extended the therapeutic efficacy of BMSC into the neuropathy induced by occupational or environmental neurotoxicants, such as *n*-hexane. The potent protective effects displayed by conditioned medium prepared from BMSC (BMSC-CM) against HD-induced apoptosis were observed in PC12 cells, which was a rat pheochromocytoma cell line and possessed properties of neurons^9^. BMSC transplantation also attenuated HD-induced neuronal damage and improved the motor performance in rats^10^. However, the mechanisms responsible for BMSC-afforded neuroprotection remain unclear.

Autophagy is an evolutionarily conserved physiological process that clears, degrades, transport and recycles cytoplasmic constituents to replenish cellular components and relieve metabolic stress^11^. Although autophagy occurs at basal levels to maintain tissue homeostasis, it could be rapidly unregulated and contributed to cell death in various pathological conditions such as hypoxia, ischemia, oxidative stress, and endoplasmic reticulum stress^12,13^. BMSC have been shown to be able to suppress activation of autophagy, therefore, mitigating the damage associated with ischemia/reperfusion injury^14^, traumatic brain injury^15^, and silicosis^16^. Although whether autophagy is dysregulated or not in *n*-hexane-induced neuropathy remains unclear, hyperactivation of autophagy is already reported in multiple toxicants-induced neurotoxicity, such as 1-Methyl-4-pheny l-1,2,3,6- tetrahydropyridine hydrochloride (MPTP), rotenone, and so on^17,18^. We therefore hypothesized that inactivation of autophagy in *n*-hexane-induced neuropathy might be responsible for BMSC transplantation-elicited neuroprotection.

To test our hypothesis, BMSC were given to HD-intoxicated rats by tail vein injection after 5 weeks of HD treatment. We found that in intravenously grafted rats, BMSC significantly reduced the levels of microtubule-associated protein 1 light chain 3-II (LC3-II) and the degradation of sequestosome-1 (p62) in the spinal cord and sciatic nerve of HD-intoxicated rats. Activation of becline 1-independent mammalian target of rapamycin (mTOR)/Unc-like kinse 1 (ULK1) pathway contributed to the inhibitory effects of BMSC on HD-induced autophagy.

## Results

### BMSC transplantation attenuates HD-induced activation of autophagy in neuron

**Figure 1:**
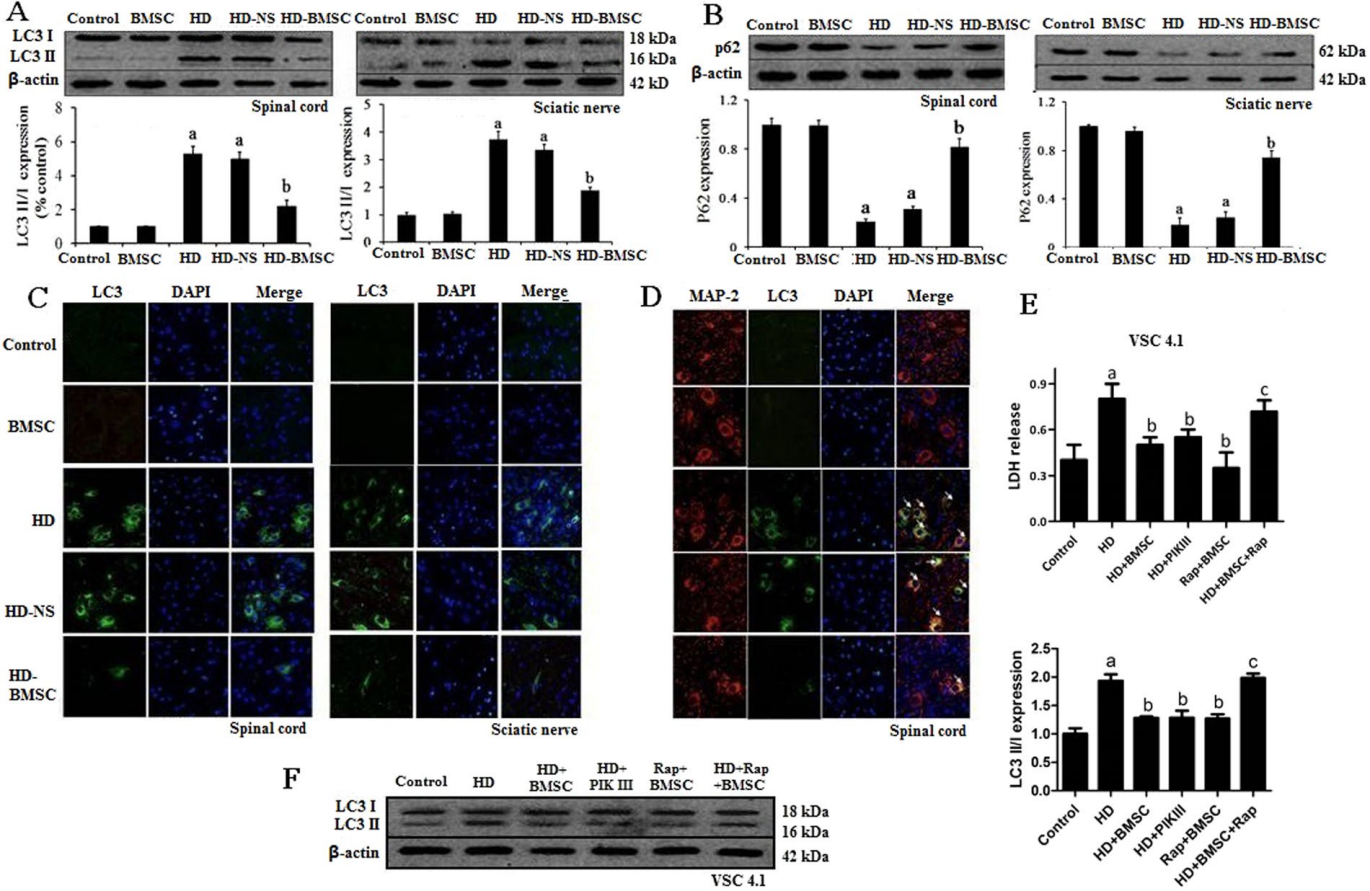
BMSC attenuates HD-induced activation of autophagy in the spinal cord and sciatic nerve of rats. (**A**,**B**) The level of LC3II (**A**) and p62 (**B**) was determined in both spinal cord and sciatic nerve of rats by western blot and the density of blots was quantified (the full-length gels were shown in Supplementary Figure S2A–D). (**C**) LC3 was stained in both spinal cord and sciatic nerve of rats and the representative images were shown. (**D**) MAP-2 and LC3 were co-stained in the spinal cord. White arrows represented MAP-2^+^/LC3^+^ cells in the fields. (**E**) Inhibition of autophagy by PIK III protects against HD-induced neurotoxicity and, meanwhile, blocks the neuroprotective effects of BMSC. VSC4.1 cells were treated with saline or HD (25 mM) for 24 h and then were treated with BMSC-CM (15%, v/v) or PIK III (20 uM) for additional 24 h. LDH analysis was used to detect the viability of VSC4.1 cell. (**F**) The level of LC3 II in VSC4.1 cells was determined in different groups and the density of blots was quantified (the full-length gels were shown in Supplementary Figure 2E). Quantified data are shown as mean ± sd. ^a^*p* < 0.05, compared with control group; ^b^*p* < 0.05, compared with HD group.

We previously reported that BMSC transplantation improved motor functional recovery and alleviates pathological damage of axon and myelination in the spinal cord and sciatic nerve of HD-intoxicated rats^10^. To determine whether autophagy is involved in BMSC-afforded protection, we detected the alterations of autophagy in HD-treated rats with or without BMSC transplantation. Western blot analyses showed a significant increase of the lipidated form of microtubule-associated protein-1 light chain 3 (LC3II), a marker for autophagy activation, in both spinal cord and sciatic nerve of HD-intoxicated rats (Fig. 1A). Consistently, the levels of the autophagosomal membrane protein, p62, were dramatically decreased in both detected tissues (Fig. 1B). Interestingly, the changes of both LC3II and p62 in HD-intoxicated rats were significantly reversed by BMSC transplantation (Fig. 1A and B), suggesting that BMSC blocked HD-induced activation of autophagy. Immunofluorescence staining using anti-LC3 antibody further confirmed this conclusion (Fig. 1C**)**. The elevated expressions of LC3 in both spinal cord and sciatic nerve of HD-intoxicated rats were significantly reduced to control level by BMSC (Fig. 1C). No significant effects of saline (HD-NS group) on HD-induced autophagic activation were observed (Fig. 1A–C).

To determine whether the inhibitory effects of BMSC on HD-induced activation of autophagy occurred in neurons, we performed double immunofluorescence staining using anti-LC3 and anti-MAP-2 antibodies in the spinal cords of rats. We found high expressions of LC3 in MAP-2-immunoreactive cells in HD-treated rats, which was significantly reduced by BMSC graft (Fig. 1D).

To determine whether inhibition of autophagic activation by BMSC was associated its neuroprotection, an *in vitro* model of HD-induced toxicity was used. Consistent with that of *in vivo*, VSC4.1 cells, a cell line of dorsal motor neurons, treated with HD displayed an increased level of LC3II. Treatment with PIK III, an inhibitor of autophagy, significantly attenuated the increase of LC3II in HD-treated VSC4.1 cells, which was associated with decrease of LDH release (Fig. 1E,F), indicating that inhibiting autophagic activation protects against HD-induced neurotoxicity. Interestingly, a comparable level of neuroprotection was detected in BMSC-CM and HD-intoxicated VSC4.1 cells (Fig. 1E). More importantly, stimulating activation of autophagy by rapamycin (Rap), a autophagy activator, abolished the protective effects of BMSC-CM against HD-induced increase of LC3II and LDH release (Fig. 1E,F), suggesting that inhibition of autophagic activation is involved in BMSC-afforded neuroprotection.

### NGF is essential for the inhibitory effects of BMSC on autophagy

**Figure 2:**
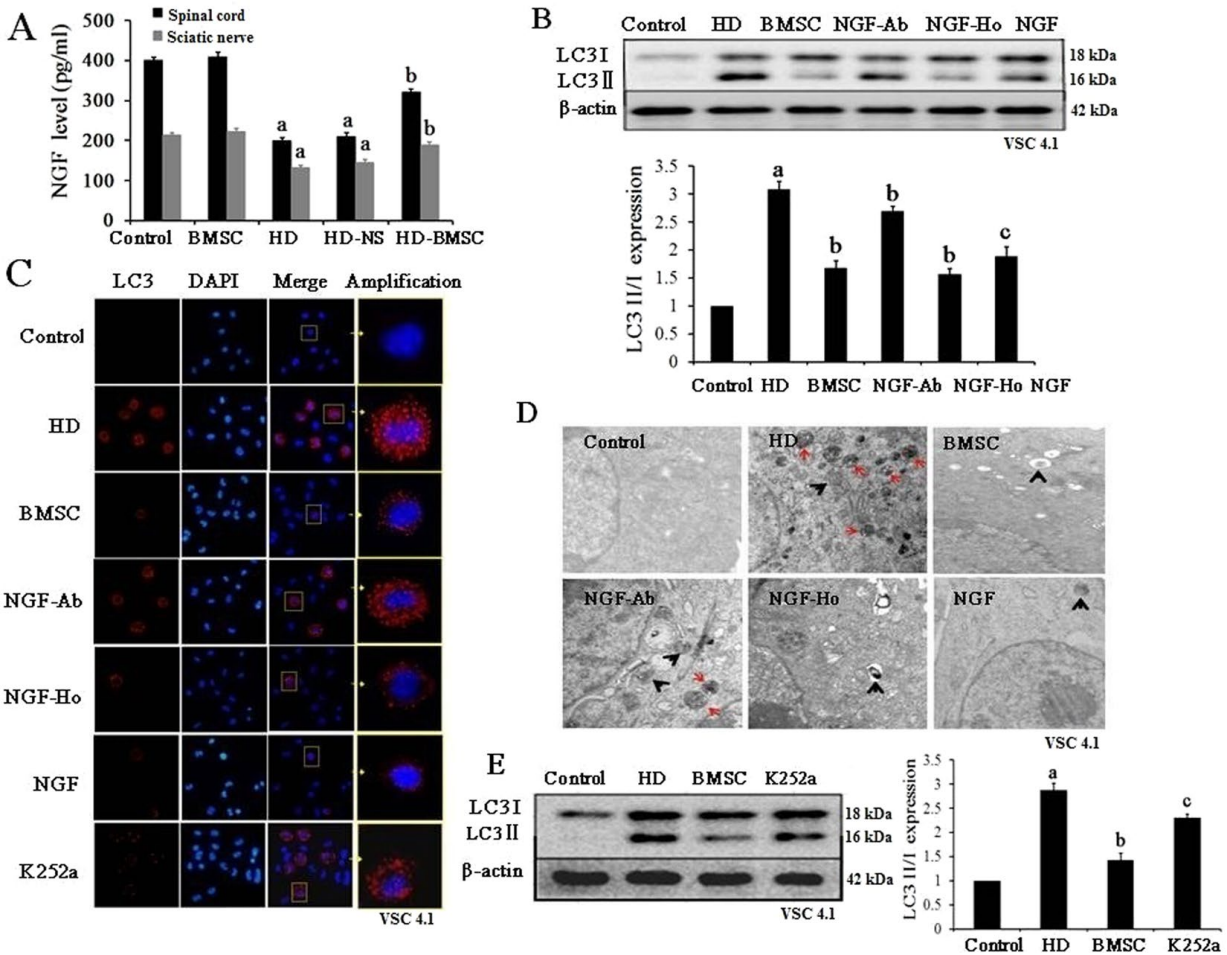
NGF is a key to mediate BMSC-afforded neuroprotection. (**A**) The protein levels of NGF were detected in the spinal cord and sciatic nerve of rats by using commercial ELISA kit. (**B**) Neutralization of NGF by using anti-NGF antibody abolishes the inhibitory effects of BMSC against HD-induced elevation of LC3II. VSC4.1 cells were treated with HD (25 mM) or saline for 24 h and then were treated with BMSC-CM (15%, v/v) or NGF (100 μM) in the presence or absence of anti-NGF antibody (10 μM) for additional 24 h. The levels of LC3 determined by western blot and the density of blots were quantified (the full-length gels were shown in Supplementary Figure S3A). (**C)** LC3 was stained in different groups and the representative images were shown. (**D**) TEM was used to detect the autophagosome in different groups. (**E**) K252a, the inhibitor of TrkA, attenuates the inhibitory effects of BMSC against HD-induced elevation of LC3II. VSC4.1 cells were treated with HD (25 mM) or saline for 24 h and then were treated with BMSC-CM (15%, v/v) with or without 1 h pre-treatment of the inhibitor of TrkA, K252a, for additional 24 h. The levels of LC3 determined by western blot and the density of blots were quantified (the full-length gels were shown in Supplementary Figure S3B). ^a^*p* < 0.05, compared with control group; ^b^*p* < 0.05, compared with HD group; ^c^*p* < 0.05, compared with BMSC-CM group.

Subsequently, we investigated the mechanisms by which BMSC attenuated activation of autophagy induced by HD. Previous study revealed that neurotrophic factor NGF, plays a key role in BMSC-elicited protection. We therefore measured the levels of NGF in BMSC-transplanted rats by using ELISA kit. We found that HD intoxication decreased the protein levels of NGF in both spinal cord and sciatic nerve of rats, which was significantly attenuated by BMSC (Fig. 2A, Supplementary Figure S1A). Consistent with increase of NGF by BMSC, TrkA, the G-protein coupled receptor of NGF were also activated in BMSC/HD-treated rats by showing an increased level of phosphorylated TrkA, compared with HD alone group (Supplementary Figure S1B).

To determine whether NGF mediates the inhibitory effects of BMSC on autophagic activation, NGF was neutralized in BMSC-CM by anti-NGF antibody (NGF-Ab group). As shown in Fig. 2B, NGF neutralization significant blocked the inhibitory effects of BMSC-CM against HD-induced increase of LC3II in VSC4.1 cells. No significant effect was observed in control IgG group (NGF-Ho group). Consistently, the decreased expression of LC3 and number of double membrane structure by BMSC-CM detected by confocal microscopy and TEM, respectively, were also blocked by anti-NGF antibody but not anti-control IgG (Fig. 2C,D). These effects were further verified by K252a, the inhibitor of TrkA, as shown by increased level of LC3II in K252a/BMSC-CM/HD-treated VSC4.1 cells (K252a group) compared with BMSC-CM/HD group (Fig. 2C,E).

### BMSC fails to interfere with expression of beclin 1, but stimulates activation of mTOR in HD-intoxicated rats

**Figure 3:**
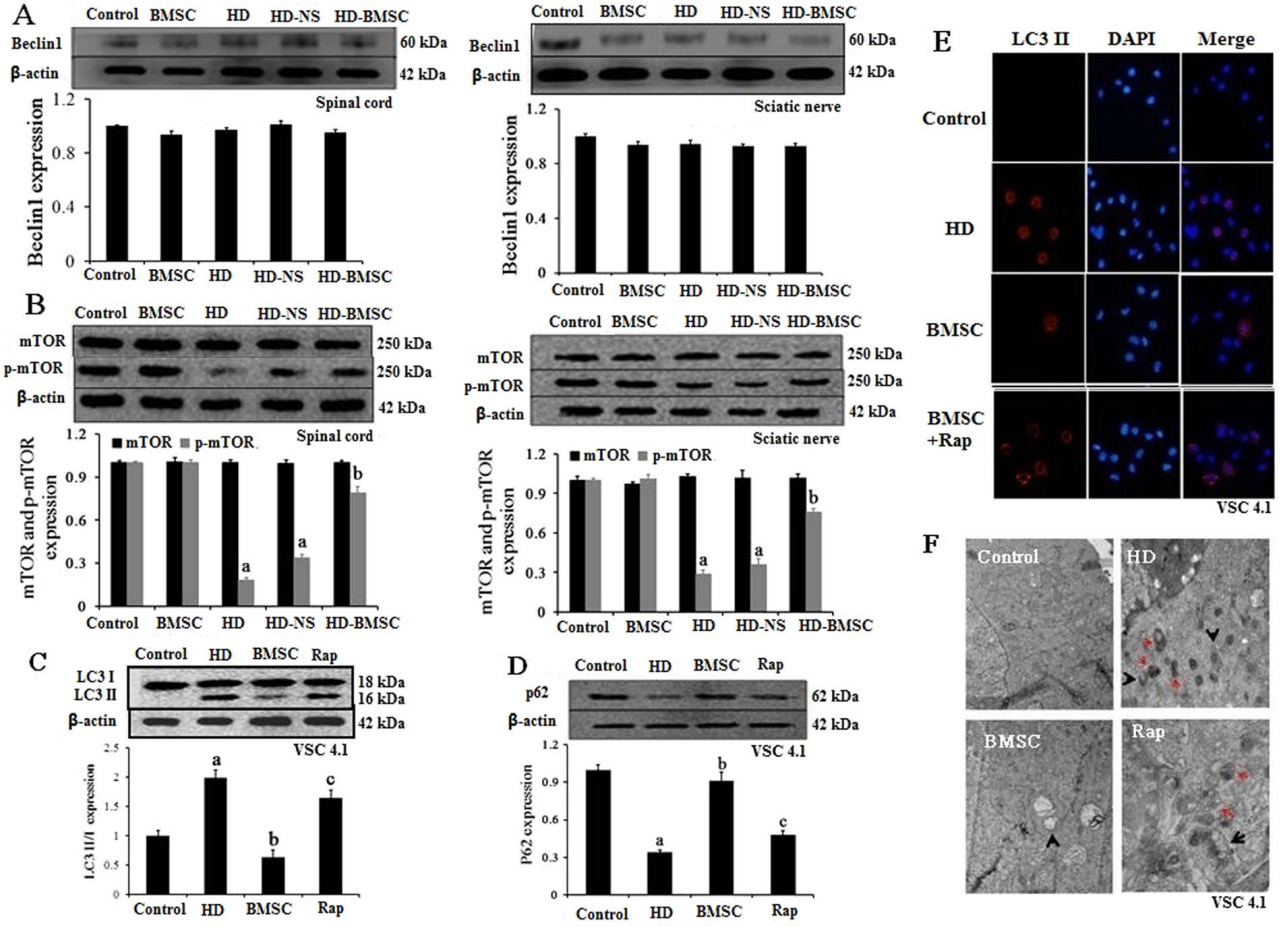
BMSC fails to interfere with the expression of Beclin 1, but stimulates activation of mTOR pathway. (**A**) The protein levels of Beclin 1 were determined in the spinal cord and sciatic nerve of rats by western blot and the density of blots were quantified (the full-length gels were shown in Supplementary Figure S4A,B). (**B**) The protein levels of mTOR and p-mTOR was determined in the spinal cord and sciatic nerve of rats by western blot and the density of blots were quantified (the full-length gels were shown in Supplementary Figure S4C,D). Quantified data are shown as mean ± SD. ^a^*p* < 0.05, compared with control group; ^b^*p* < 0.05, compared with HD group. (**C**) The inhibitors of mTOR (Rapamycin, Rap) attenuate the inhibitory effects of BMSC against HD-induced elevation of LC3II. VSC4.1 cells were treated with HD (25 mM) or saline for 24 h and then were treated with BMSC-CM (15%, v/v) in the presence or absence of Rap for additional 24 h. The levels of LC3 were determined by western blot and the density of blots was quantified (the full-length gels were shown in Supplementary Figure S4E). (**D**) Rap attenuates the inhibitory effects of BMSC against HD-induced degradation of p62 in VSC4.1 cells (the full-length gels were shown in Supplementary Figure S4F). (**E**) LC3II was staining in different groups and the representative images were shown. (**F**) TEM was performed in different groups and the representative images were shown. Data represent mean ± SD. ^a^*p* < 0.05, compared with control group; ^b^*p* < 0.05, compared with HD group; ^c^*p* < 0.05, compared with BMSC-CM group.

Beclin 1 is an important autophagy regulator playing several different roles along the autophagic process^19^. We therefore detected the expressions of Beclin 1 in the spinal cord and sciatic nerve of HD-intoxicated rats with or without BMSC transplantation. No significant difference was observed in Beclin 1 expression in different groups (Fig. 3A), suggesting that Beclin 1 is not involved in the regulatory effects of BMSC on autophagy.

Strong evidence suggests that mTOR plays a pivotal role in regulating autophagy^20^. Immunoblot analyses revealed a significant decrease of activation of mTOR in the spinal cord and sciatic nerve of HD-intoxicated rats by showing reduced levels of phosphorylated mTOR, compared with vehicle controls (Fig. 3B). Interestingly, the reduced levels of phosphorylated mTOR were recovered by BMSC transplantation (Fig. 3B), suggesting that BMSC stimulates activation of mTOR.

### Activation of mTOR is involved in BMSC-induced inhibition of autophagy

The role of mTOR in BMSC-inhibited autophagic activation was subsequently determined by using inhibitors. As seen in Fig. 3C, HD exposure markedly increased the levels of LC3II in VSC4.1 cells, which was significantly reduced by BMSC-CM, consistent with that of *in vivo*. Interestingly, the inhibitory effects of BMSC-CM on HD-induced increase of LC3II were blocked by pre-treatment with inhibitors of mTOR, Rap (Fig. 3C). In agreement, the inhibitory effects of BMSC-CM against HD-induced reduction of p62 were also mitigated by Rap (Fig. 3D), indicating that mTOR is involved in BMSC-elicited inhibition of autophagic activation. Immunofluorescence staining using anti-LC3 antibody and TEM analyses further supported this conclusion. As seen in Fig. 3E and F, compared with HD/BMSC-CM group, a recovery of LC3 density and double membrane structures in HD and BMSC-CM-treated cells was observed in the presence of Rap.

### BMSC inhibits activation of ULK1 through a mTOR-dependent manner

**Figure 4:**
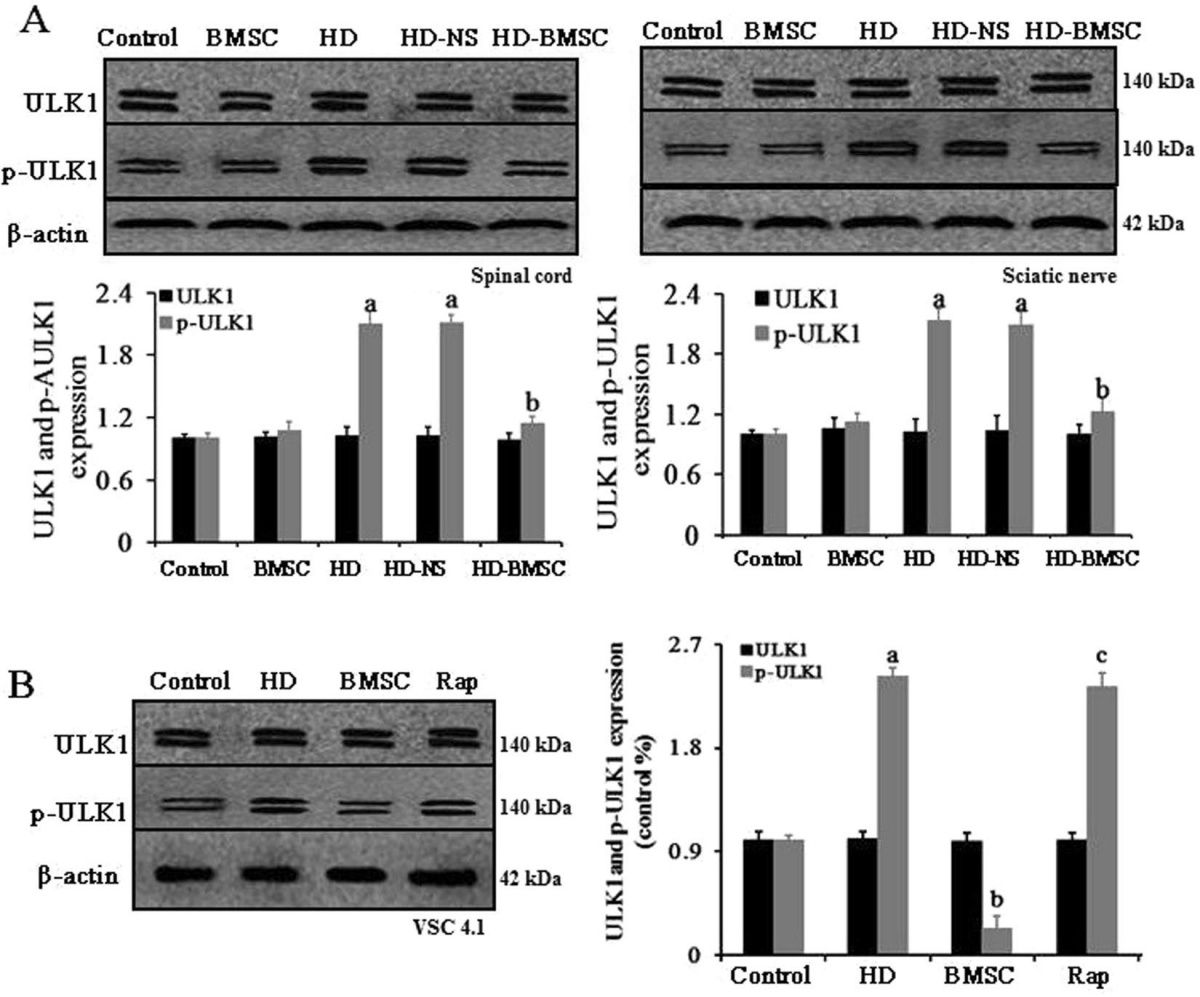
mTOR-dependent activation of ULK1 contributes the anti-autophagic effects of BMSC. (**A**) The effects of BMSC transplantation on the levels of ULK1 and p-ULK-1 in the spinal cord and sciatic nerve of HD-intoxicated rats were detected using western blot and the density of blots was quantified (the full-length gels were shown in Supplementary Figure S5A,B). (**B**) Rapamycin abolished the inhibitory effects of BMSC against HD-induced phosphorylation of ULK1. VSC4.1 cells were treated with HD (25 mM) or saline for 24 h and then were treated with BMSC-CM (15%, v/v) in the presence or absence of Rap for additional 24 h. The levels of ULK1 and p-ULK-1 were determined by Western blot and the density of blots was quantified (the full-length gels were shown in Supplementary Figure S4C). ^a^*p* < 0.05, compared with control group; ^b^*p* < 0.05, compared with HD group; ^c^*p* < 0.05, compared with BMSC-CM group.

The ULK1, a downstream signal of mTOR, plays an essential role in the initiation of autophagy^21^. To investigate whether ULK1 is involved in BMSC-elicited inhibition of autophagy, the activation of ULK1 was determined in HD-intoxicated rats with BMSC transplantation. We found that the levels of phosphorylated ULK1 in the spinal cord and sciatic nerve of HD-intoxicated rats were significantly increased, which was markedly decreased in BMSC-grafted rats, indicating that BMSC blocks HD-induced activation of ULK1 (Fig. 4A).

To determine whether mTOR mediates the inhibitory effects of BMSC against HD-induced ULK1 activation, VSC4.1 cells were used. Interestingly, BMSC-CM-elicited inhibition of ULK1 phosphorylation was markedly recovered by Rap (Fig. 4B).

## Discussion

In this study, we demonstrated that BMSC transplantation inhibited activation of autophagy in HD-intoxicated rats through beclin 1-independent activaton of mTOR pathway, which might be responsible for BMSC-afforded neuroprotection. The salient features of our findings are: (1) BMSC reduced the levels of LC3-II and the degradation of p62 in HD-intoxicated rats; (2) BMSC had no effect on Beclin 1, but promoted activation of mTOR/ULK1 pathway; (3) Inhibition of mTOR pathway blocked the inhibitory effects of BMSC on autophagic activation.

Autophagy refers to a highly conserved process by targeting bulk intracellular components for lysosomal degradation. Numerous reports revealed that dysregulation of autophagy may serve as a major mediator of cell death in pathological situations as shown by cell death can be prevented by pharmacological inhibition of autophagy or by knockdown of autophagy genes. Enhanced autophagy has been implicated in various neurological conditions including cerebral ischemia and head and spinal cord injury^22–24^. However, whether autophagy is involved in *n*-hexane-induced neuropathy remain undefined. Since autophagic activation has been shown to regulate axon degeneration in previous study^25^, we hypothesized that autophagic activation might contribute to *n*-hexane-induced neurotoxicity. In agreement, in the present study, we observed elevated autophagic activation in HD-intoxicated rats by showing increased LC3II level and p62 degradation in the spinal cord and sciatic nerve, the target tissues of *n*-hexane-induced neuropathy. Moreover, inhibition of autophagy by PIK III strongly reduced the neurotoxic effects of HD, indicating that autophagic activation contributes to HD-induced neurotoxicity. Chu *et al*. reported that 1-methyl-4-phenylpyridinium (MPP^+^), a parkinsonian neurotoxin, induces a robust increase of autophagic vacuoles in SH-SY5Y neuroblastoma cells and primary dopaminergic midbrain neurons, which is accompanied by a significant cell loss^26^. In tri-ortho-cresyl phosphate-induced delayed neuropathy in hens, increased number of autophagsomes and levels of LC3II in spinal cord motor neurons were also detected^27^, being consistent with our current findings. However, in Song *et al*.’s study, an increased level of p62 in sciatic nerve of HD-intoxicated rats was observed, which might be represent an compensatory effect for the degradation of neuroinflamments since they detected p62 at early stage of HD-induced neuropathy^28^. Despite of this, our findings suggest that autophagic activation is actively involved in the pathological process in environmental toxins-induced toxicity.

The involvement of autophagy in HD-induced toxicity prompts us to investigate whether BMSC are capable of inhibiting autophagy, thereby affording neuroprotective effects. Interestingly, we found that BMSC transplantation significantly attenuated increase of LC3II, formation of autophagosome and degradation of p62 in HD-intoxicated rats, which was associated with recovery of motor performance and electrophysiological activities. In agreement with our results, BMSC-afforded neuroprotection via autophagic inhibition was also observed in rat models of ischemia/reperfusion injury^14^, acute lung injury^29^ and traumatic brain injury^15^. Notably, different from previous reports, BMSC transplantation in the present study was performed in a post-treatment regimen, i.e., after 5 weeks of HD intoxication, which is significant from a clinic point of view.

Mechanistically, the most critical question to address is how BMSC inhibits autophagic activation induced by HD. The process of autophagy is tightly regulated by a group of evolutionarily conserved ATG genes, such as ATG^30^. Among them, beclin-1 is essential and has been shown to act in the induction step prior to autophagy^19^. In addition to genetic manipulation, signaling pathways, such as PI3K/Akt/mTOR, are also important to autophagy in response to cellular physiological conditions and environmental stress^20^. One particularly interesting observation of the present study is the discovery that the inhibitory effects of BMSC on HD-induced activation of autophagy were beclin 1-independent activation of mTOR pathway. HD-intoxicated rats with or without BMSC transplantation displayed no changes of beclin 1 expression in both spinal cord and sciatic nerve. By contrast, the decreased activation of mTOR pathway induced by HD was recovered by BMSC transplantation by showing increased phosphorylation of mTOR compared with HD alone group. Moreover, inhibition of mTOR using pharmacological inhibitor blocked the inhibitory effects of BMSC against HD-induced activation of autophagy. ULK1 was further recognized as the downstream signal of mTOR since the inhibition of BMSC on HD-induced ULK1 activation was blocked by mTOR inhibitor, Rap. Although there is no report for the inhibitory effects of BMSC on autophagic activation in previous studies, several compounds have been shown to induce cell damage through a beclin 1-independent activation of autophagy. Grishchuk *et al*. found that saturosporine, a potent pro-apoptotic agent, induces apoptosis in cortical neurons through a beclin 1-independent activation of autophagy^31^. Beclin 1-independent activation of autophagy was also found in Hela cells treated with Z18 (a Bcl-X_L_/Bcl-2 inhibitor)^32^ and midbrain primary dopaminergic neurons intoxicated with MPP^+ ^^33^. Our results indicated that BMSC protected against *n*-hexane-induced overautophagy through beclin 1-independent inhibition of mTOR signaling pathway. In traditional neurodegenerative disorders, such as Alzheimer disease (AD) and Parkinson’ s disease (PD), autophagy was likely to be inhibited and BMSC exerted neuroprotection by enhancing autophagic activation^34,35^. In contrast, in some environmental toxicants-induced neurotoxicity, autophagy appeared to be activated^36–38^ and BMSC might elicit neuroprotective effects by dampening autophagic activation. Although the exact mechanism remains unclear, the discrepancy among different pathological conditions might be one of the reasons. In neurodegenerative diseases, the deposition of specific proteins, such as α-synuclein and β-amyloid, was usually observed, which could be ameliorated by BMSC-mediated activation of autophagy. However, in toxicants-induced neurotoxicity, the target proteins were usually degraded^39,40^. Neurofilaments (NFs), the important protein for maintenance of axon diameter, were the key targets of n-hexane-induced neuropathy. The levels of NFs were significantly decreased in nerve tissues in rats treated with 2,5-hexanedione, the active metabolite of n-hexane^41,42^. Inhibition of autophagy by BMSC might reduce the degradation of NFs and therefore protected rats against 2,5-hexanedione-induced neurotoxicity.

The beneficial effects of transplanted MSCs were originally thought to be mediated by MSCs migrating to the site of injury and differentiating into specialized cells. However, only a small proportion of transplanted MSCs were observed to be truly migrated to and survived in damaged host tissue^43^. Recently, growing evidence has indicated that stem cells exert their beneficial effects by secreting neurotrophic factors (NTs), cytokines and chemokines etc. around the damaged tissue, mainly through a paracrine mechanism^44^. NGF is the best-known member of a family of NTs and it was revealed that NGF plays a key role in BMSC-elicited protection^45^. In the present study, we found that BMSC significantly upregulated the decreased protein levels of NGF in both spinal cord and sciatic nerve of rats exposed to HD. Moreover, after NGF was neutralized in BMSC-CM by anti-NGF antibody in the intervention experiment *in vitro*, the inhibitory effects of BMSC-CM against HD-induced increase of LC3II were significantly blocked in VSC4.1 cells. Consistently, the inhibitory effects of BMSC-CM were also significantly mitigated in the presence of K252a, an inhibitor of TrkA as a NGF receptor. It has been documented that NGF can activate PI3K/Akt signaling pathway via combining TrkA while mTOR is its downstream signaling molecule and is activate by the signaling pathway^46^. Our results indicate that NGF may be a key regulator for the inhibitory effects of BMSC on HD-induced excessive autophagy via Akt/mTOR signaling pathway.

In summary, this study indicates that BMSC transplantation is capable of protecting against HD-induced neuropathy. In this process BMSC attenuates motor deficits and pathological damage and affect beclin 1-independent activation of autophagy in HD-intoxicated rats, such as blocking expression of LC3II, increasing level of p62, reducing the number of autophagysome and stimulating mTOR pathway as well as inhibiting activation of ULK1. Our findings provide a novel insight for the mechanisms of BMSC in combating HD-induced neuropathy. Because n-hexane can induce peripheral neuropathy, occupational health and safety measures for preventing n-hexane exposure should be the best solution to the toxicant poisoning. However, if n-hexane is exposed and serious peripheral neuropathy is induced, BMSC transplantation may be a potential therapeutic avenue.

## Material and Methods

### BMSC culture

BMSC were isolated as previously described^9^. Briefly, 60~90 g Sprague Dawley (SD) rats (Laboratory Animal center, Dalian Medical University, China) were euthanatized by cervical dislocation and bone marrow was flushed from the femurs and tibias with PBS. A single-cell suspension was obtained by sieving and seeded in Dulbecco’s modified eagle medium (DMEM)-low glucose (Gibco, USA) with 10% fetal bovine serum (FBS, HyClone, USA), 100 U/ml penicillin and 100 μg/ml streptomycin (Beyotime, China) at 37 °C in 95% humidified air. Twenty-four hours later, the media were replaced to remove non-adherent cells. The characteristic surface makers (CD29, CD45 and CD90) were detected by flow cytometry to identify BMSC^34^. Adipogenic and osteogenic differentiation ability of BMSCs was also evaluated after induction by specific media Oil Red O and Alizarin Red S staining (Cyagen, China)^47^. All the experiments were done within 5th passage.

### Preparation of BMSC-CM

BMSC-CM were prepared by the established method by our lab^10^. In brief, confluent BMSC, at 3~5 passage, were transferred to fresh medium for 24 h after washing with phosphate-buffered saline (PBS). The culture supernatants were collected and then centrifuged at 1,500 rpm for 10 minutes at 4 °C. The supernatants were transferred to a centrifugal column with a 3 kDa cut-off (Millipore, Billerica, MA, USA) and centrifuged at 3,500 rpm for 45 minutes at 4 °C. The BMSC-CM was desalted according to the manufacturer’s protocol and then was sterilized by filtration with 0.22 μm membrane. Finally, BMSC-CM was prepared for subsequent experiments.

### Animal treatment and tissue preparation

**Figure 5:**
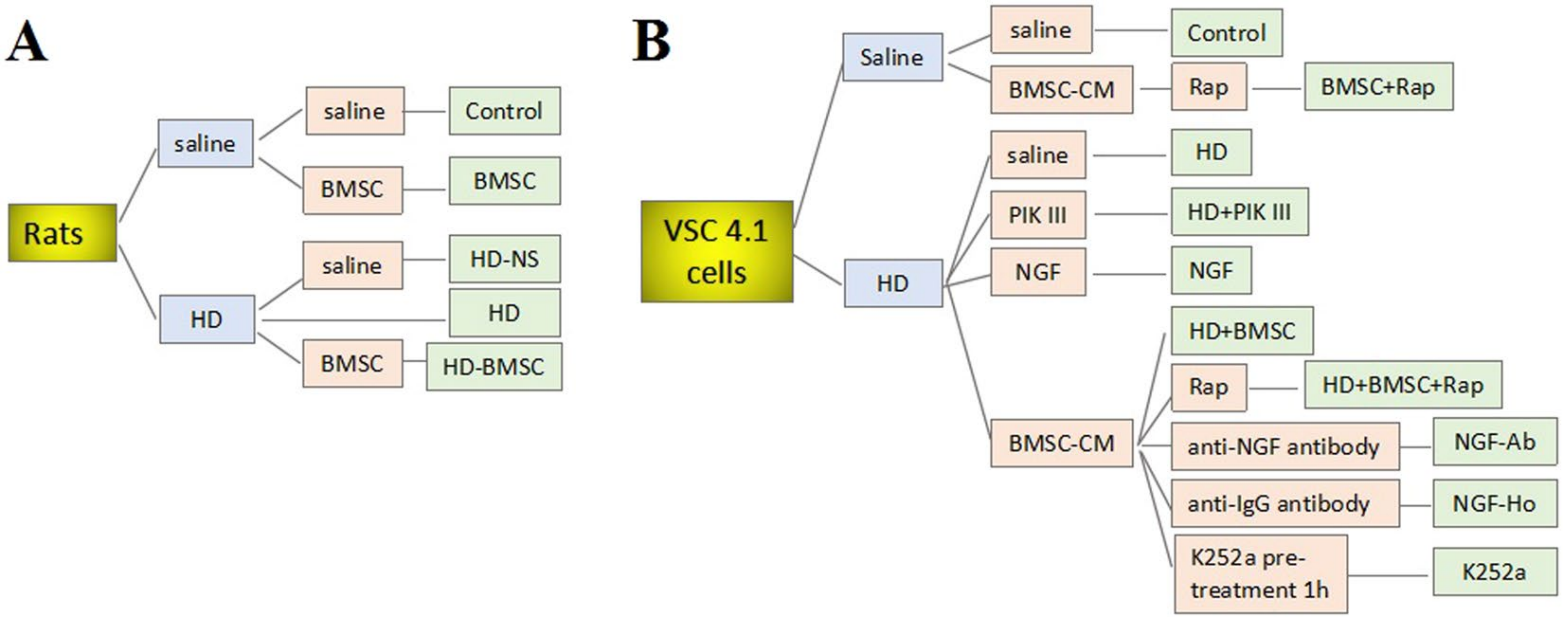
Schematic diagram of experimental design. (**A**) The schematic diagram of experimental design about rats. After 7-day acclimatization, fifty rats were randomly divided into 5 groups (n = 10 for each group). Rats were treated with HD (400 mg/kg, i.p) or saline for 5 consecutive weeks (five times per week) and then received BMSC or saline by tail vein injection (additional 5 weeks later, rats were killed). (**B**) The schematic diagram of experimental design about VSC4.1 cells. Prepared VSC4.1 Cells were treated with HD (25 mM) or saline for 24 h and then were treated with BMSC-CM (15%, v/v) in the presence or absence of some pathway inhibitors or activators.

Animals were treated as previously described^10^. Adult male SD rats (200∼230 g) were purchased from the Experimental Animal Center of Dalian Medical University and housed with sufficient drinking water and food. The animal room was maintained at approximately 22 °C and 50% relative humidity with a 12 h light-dark cycle. After 7-day acclimatization, fifty rats were randomly divided into 5 groups (n = 10 for each group). HD-BMSC and HD-NS group were treated with HD (400 mg/kg, intraperitoneal injection (i.p)) for 5 consecutive weeks (five times per week) and then were transplanted with 5 × 10^7^/kg BMSC^48^ and 0.9% saline (NS), respectively, by tail vein injection (i.v). Additional 5 weeks later, rats were killed by cervical decapitation. The spinal cords and sciatic nerves were quickly dissected and frozen in liquid nitrogen before storing them at −80 °C. Control group received saline alone for 10 weeks. BMSC group received saline for 5 weeks, and then transplanted with BMSC by tail i.v (additional 5 weeks later, rats were killed.). HD group were treated with HD for 5 weeks, and then were killed. HD was dissolved in NS and administered at 3 ml/kg body weight/dose. The corresponding control group rats received an equivalent volume of NS by i.p. Experiments were performed in accordance with the Animal Guideline of Dalian Medical University. All experimental protocols were approved by and in agreement with the Ethical Committee of Dalian Medical University, approval number: CXK (Liao) 2015–2003). The schematic diagram of experimental design about rats was showed in Fig. 5A.

### VSC4.1 cell culture and treatment

VSC4.1 motor neurons were maintained in DMEM medium containing 15 mM HEPES, pyridoxine NaHCO_3_ (Sigma, St. Louis, Missouri, USA), 2% Sato’s components, 1% penicillin and streptomycin (Beyotime, Shanghai, China) as well as 15% heat-inactivated FBS (Hyclone, Logan, UT, USA) in poly-L-ornithine coated 7.5 cm^2^ flasks. Cells were grown at 37 °C in the incubator with 5% CO_2_ and full humidity.

Cells were redistributed with DMEM containing 2% FBS at density of 10^6^ cells in 75-cm^2^ flasks and 2.5–5 × 10^4^ cells/well in 6-well plates for 24 hours, respectively. Prepared Cells were treated with HD (25 mM) or saline for 24 h and then were treated with BMSC-CM (15%, v/v) in the presence or absence of some pathway inhibitors or activators, containing PIK III (20 μM), Rap (20 μM), anti NGF antibody (10 μM), anti IgG antibody (10 μM), NGF (100 μM) and K252a (20 μM), for another 24 h. Cells cultured in DMEM (containing 2% FBS) with equal amount of saline were used as the negative control. Schematic diagram of experimental design about VSC 4.1 cells was showed in Fig. 5B.

### Lactate dehydrogenase (LDH) release assay

Cell viability was assayed by measuring the release of LDH using a commercial kit according to the manufacturer’s instructions. Brief, cells were also seeded in 96-well plates and then were washed with PBS one time when they reached approximately 90% confluence. Then, cells were treated as described above. Lastly, the OD_490_ was measured by microplate spectrophotometer (Thermo Electron Corporation, Waltham, MA, USA). A curve of LDH release was obtained, ultimately.

### TEM analysis

To determine localization Autophagic vesicles in VSC4.1 cells, transmission electron microscopy was used. In brief, cells were pre-fixed in 2.5% glutaraldehyde, washed with PBS three times, then were post-fixed in 1% osmium tetroxide for 1.5 h, dehydrated in graded ethanol, and embedded in epoxy resin. Polymerization was performed at 80 °C for 24 h. Blocks were cut on a Reichert ultramicrotome into ultrathin sections (70 nm), which were poststained with uranylacetate and lead citrate, and viewed under an electron microscope (JEOL, Japan).

### Western blot

Tissues and cells were homogenized in ice-cold RIPA Tissue Protein Extraction Reagent (Beyotime, China) supplemented with 1% proteinase inhibitor mix. The proteins were separated by SDS-PAGE and then electrotransferred to PVDF membrane (Millipore, France). The membrane was incubated with appropriate primary antibodies overnight at 4 °C. Antibodies used were NGF (1:500, Cell Signaling Technology, USA), TrkA, p-TrkA, Akt, p-Akt, Beclin1 (ser-473), LC3 (1:1000, sigma, USA), p62 (ser-136) (1:500, Abcam, USA), mTOR, p-mTOR (1:1000, Abcam, USA), ULK1, p-ULK1 (ser 757) (1:1000, Cell signaling Technology, USA) and β-actin (1:500, ZS-Bio, China). Immunoreactivity was visualized by second horseradish peroxidase-conjugated antibody (1:5000, Sigma, USA) and enhanced chemoluminescence (Beyotime, China). Quantified densitometric analysis was performed with UVP BioSpectrum Multispectral Imaging System (Ultra-Violet Products Ltd. USA). The quantification of Western blot were obtained from three independent experiments with triplication.

### Immunofluorescence staining

The frozen sections (10 μm) and VSC4.1 cells were permeabilized with 0.3% Triton X-100 for 15 min, and blocked with donkey serum albumin (1:50, Abbkine, USA) for 1 h at room temperature. Specimens were subsequently incubated with primary antibodies against LC3 (1:100) overnight at 4 °C, then washed with PBS three times, and incubated with Alexa-Fluor 594 conjugated donkey anti-rabbit IgG secondary antibody (1:500, West Grove, PA, USA) for 1 h at room temperature. After being washed, the cells were treated with DAPI for 5 min and analyzed under a fluorescence microscope (Olympus, Japan).

### Confocal double-label immunofluorescence

The frozen sections (10 μm) were immunoblocked with 10% donkey serum in 5% BSA for 1 h and then incubated with mouse monoclone anti-MAP2 antibody (1:500), overnight at 4 °C. On the second day, the sections were washed by PBS for 3 times before incubation with polyclonal rabbit anti-LC3 antibody (1:100) overnight at 4 °C. The double-label immunofluorescence pictures were taken under the confocal microscope by using Alexa-488 (green) and Alexa-594 (red) conjugated secondary antibodies (1:500). Finally, the sections were analyzed under a fluorescence microscope (Olympus, Japan).

### Statistical analysis

All results were expressed as mean ± SD, and the statistical analysis was performed with one-way analysis of variance (ANOVA), followed by LSD test, which was performed using SPSS 13.0 statistical software. The p-values less than 0.05 were considered to be significant.

## Electronic supplementary material

Supplementary Figures
