# Supplementary material for: Bone marrow mesenchymal stem cells protect against n-hexane-induced neuropathy through beclin 1-independent inhibition of autophagy
Source: Sci Rep. 2018 Mar 14;8:4516. doi: 10.1038/s41598-018-22857-x (PMC5852116; doi:10.1038/s41598-018-22857-x)
Supplement: Supplementary file 1 — Supplementary Figures [file 41598_2018_22857_MOESM1_ESM.pdf]

Bone marrow mesenchymal stem cells protect against *n*-hexane-induced neuropathy through beclin 1-independent inhibition of autophagy

Jie Hao[1*], Shuangyue Li[1*], Xiaoxia Shi[1*], Zhiqiang Qian[1], Yijie Sun[1], Dunjia Wang[1], Xueying Zhou[1], Hongxin Qu[1], Shuhai Hu[2], Enjun Zuo[2], Cong Zhang[3], Liyan Hou[1], Qingshan Wang[1†], Fengyuan Piao[1†]

[1] Department of Occupational and Environmental Health, Dalian Medical University, Dalian, Liaoning 116044, China

[2] College of Stomatology, Dalian Medical University, Dalian, Liaoning 116044, China

[3] Department of Nutrition and Food Safety, Dalian Medical University, Dalian, Liaoning 116044, China

[*]Jie Hao, Shuangyue Li and Xiaoxia Shi as co-first authors contributed equally to this work.

[†]Corresponding authors: Fengyuan Piao, E-mail: piaofengyuan353@163.com; Co-corresponding authors: Qingshan Wang, E-mail: wangq4@126.com

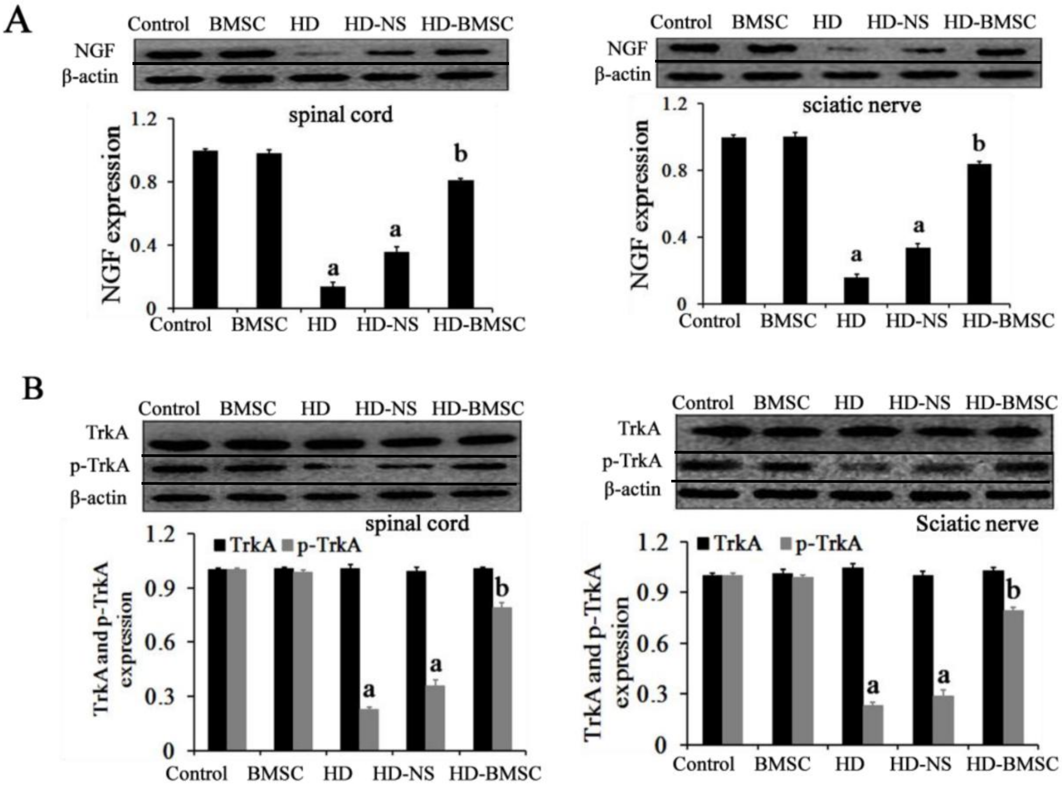

Supplementary Figure S1. BMSC graft elevates the level of NGF in the spinal cord and sciatic nerve of HD-intoxicated rats. A, The protein level of NGF was assayed by western blot and the density of blots was quantified (the full-length gels were shown in Supplementary Figure 6A, 6B). B, The levels of TrkA and p-TrkA by western blot and the density of blots was quantified (the full-length gels were shown in Supplementary Figure 6C, 6D). $^{a}p < 0.05$, compared with control group; $^{b}p < 0.05$, compared with HD group; $^{c}p < 0.05$, compared with BMSC-CM group.

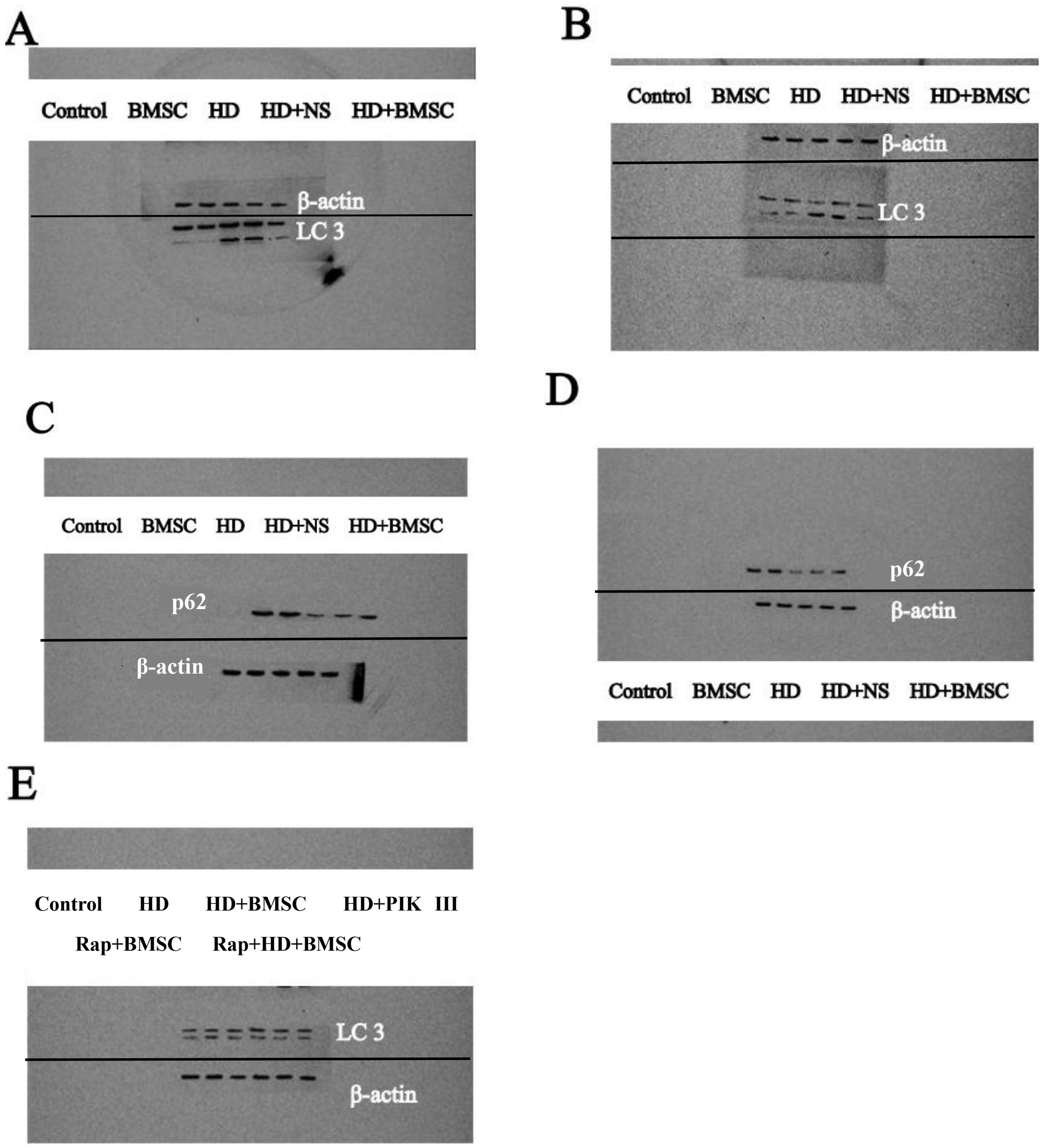

Supplementary Figure S2. The full-length gels for Figure 1. A,B, The full-length Western blot gels of LC3 and its β-actin in spinal cord (A) and sciatic nerve (B) of HD-intoxicated rats with or without BMSC transplantation. C,D, The full-length Western blot gels of p62 and its β-actin in spinal cord (C) and sciatic nerve (D) of HD-intoxicated rats with or without BMSC transplantation. E, The full-length Western blot gels of LC3 and β-actin in HD-intoxicated VSC4.1s with autophagy inhibitor PIKIII or autophagy activator Rap.

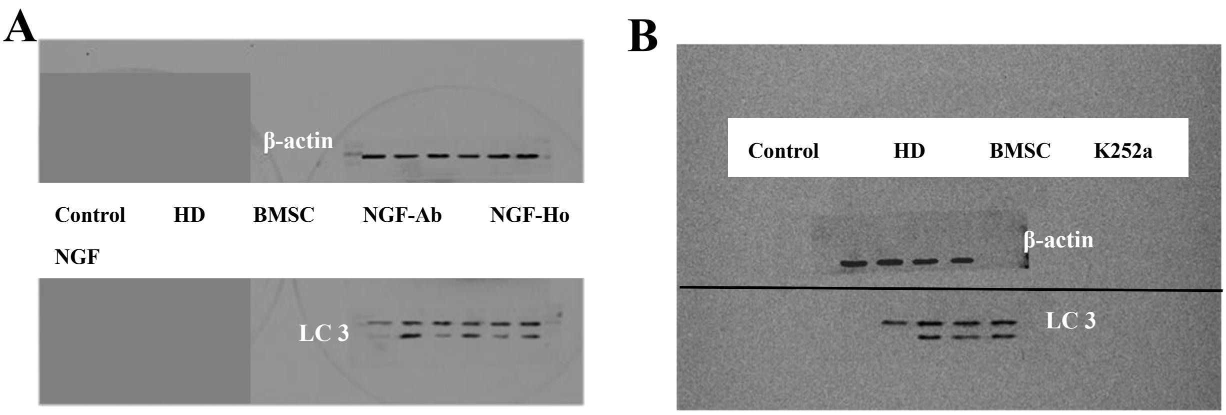

Supplementary Figure S3. The full-length gels for Figure 2. A, The full-length Western blot gels of LC3 and its β-actin in HD-intoxicated VSC4.1 cells with NGF-Ab or NGF. B, The full-length Western blot gels of LC3 and its β-actin in HD-intoxicated VSC4.1 cells with or without K252a.

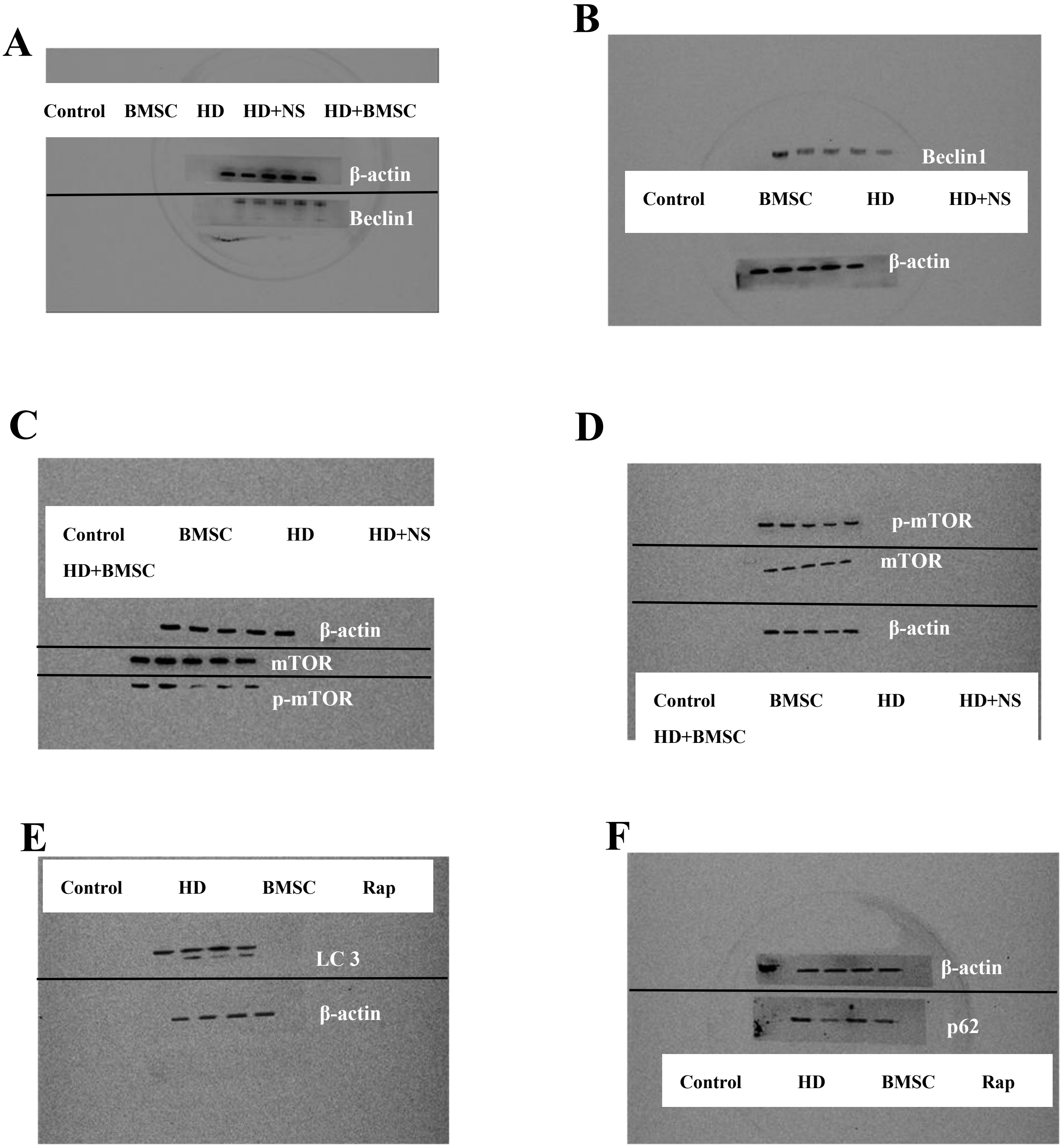

Supplementary Figure S4. The full-length gels for Figure 3. A,B, The full-length Western blot gels of Beclin1 and its β-actin in spinal cord (A) and sciatic nerve (B) of HD-intoxicated rats with or without BMSC transplantation. C,D, The full-length Western blot gels of mTOR, p-mTOR and its β-actin in spinal cord (C) and sciatic nerve (D) of HD-intoxicated rats with or without BMSC transplantation. E, F, The

full-length Western blot gels of LC3 (E), P62(F) and its β-actin in HD-intoxicated VSC4.1 cells with or without Rap.

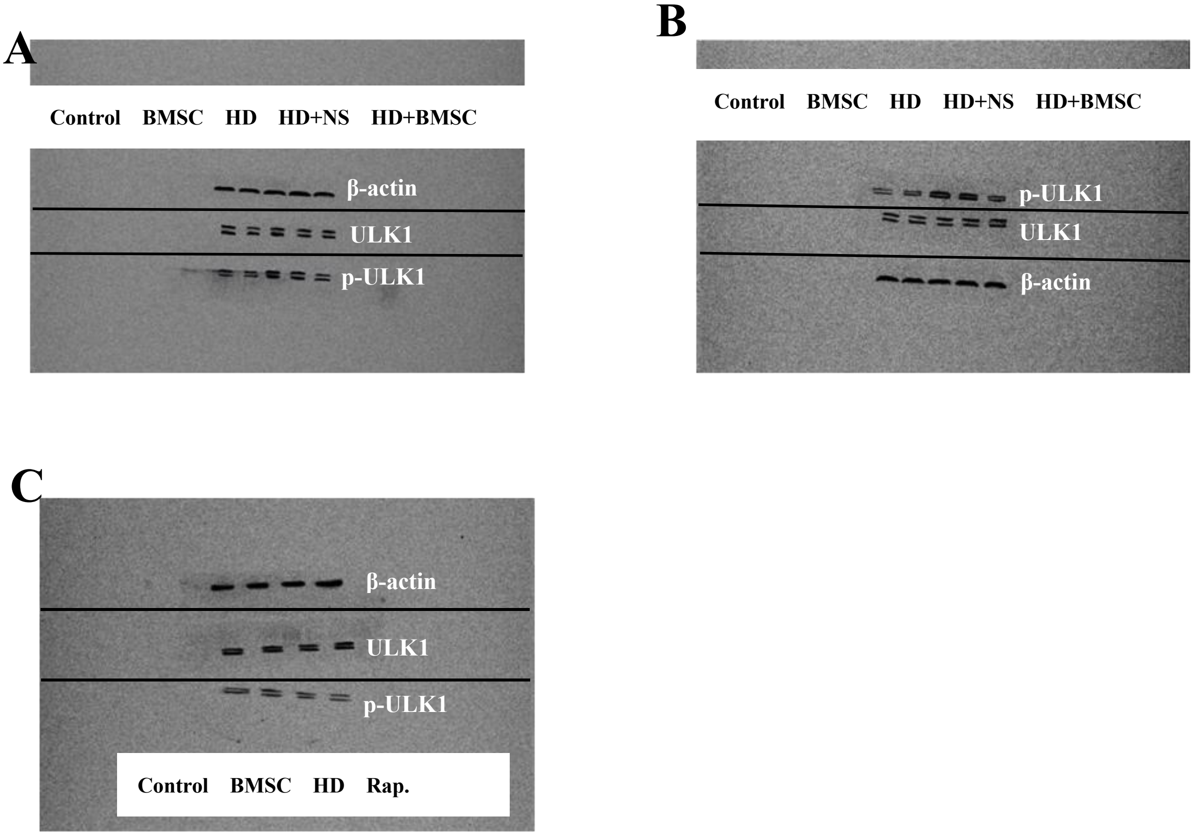

Supplementary Figure S5. The full-length gels for Figure 4. A,B, The full-length Western blot gels of ULK1, p-ULK-1 and its β-actin in spinal cord (A) and sciatic nerve (B) of HD-intoxicated rats with or without BMSC transplantation. C, The full-length Western blot gels of ULK1, p-ULK-1 and its β-actin in HD-intoxicated VSC4.1s with or without Rap.

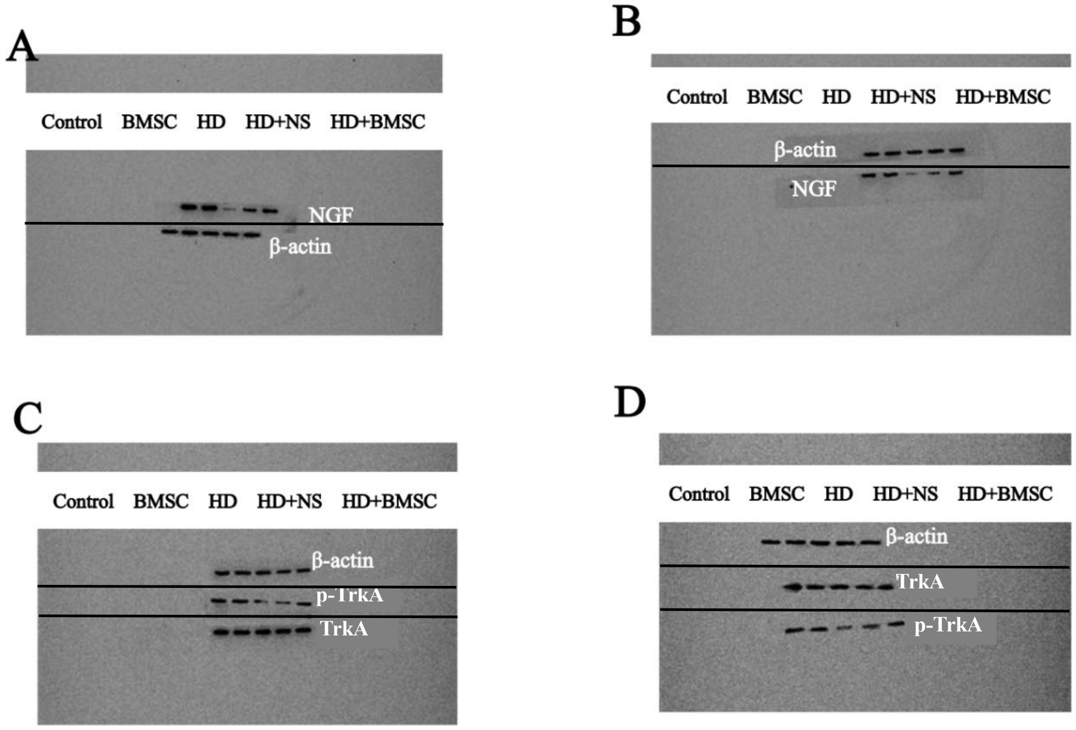

Supplementary Figure S6. The full-length gels for Figure S1. A,B, The full-length Western blot gels of NGF and its β-actin in spinal cord (A) and sciatic nerve (B) of HD-intoxicated rats with or without BMSC transplantation. C,D, The full-length Western blot gels of TrkA, p-TrkA and its β-actin in spinal cord (C) and sciatic nerve (D) of HD-intoxicated rats with or without BMSC transplantation.
